# Multilayer WS_2_ for low-power visible and near-infrared phototransistors

**DOI:** 10.1186/s11671-024-04000-0

**Published:** 2024-03-25

**Authors:** Aniello Pelella, Kimberly Intonti, Ofelia Durante, Arun Kumar, Loredana Viscardi, Sebastiano De Stefano, Paola Romano, Filippo Giubileo, Hazel Neill, Vilas Patil, Lida Ansari, Brendan Roycroft, Paul K. Hurley, Farzan Gity, Antonio Di Bartolomeo

**Affiliations:** 1 Department of Science and Technology, University of Sannio, Via De Sanctis 59/A, 82100 Benevento, Italy; 2 Department of Physics “E. R. Caianiello”, University of Salerno, Via Giovanni Paolo II, 84084 Fisciano, Salerno Italy; 3 CNR-SPIN Salerno, Via Giovanni Paolo II, 84084 Fisciano, Italy; 4 Tyndall National Institute, University College Cork, Lee Maltings, Dyke Parade, Cork, T12 R5CP Ireland; 5 School of Chemistry, University College Cork, Cork, Ireland

## Abstract

Mechanically exfoliated multilayer WS_2_ flakes are used as the channel of field effect transistors for low-power photodetection in the visible and near-infrared (NIR) spectral range. The electrical characterization as a function of the temperature reveals devices with n-type conduction and slightly different Schottky barriers at the drain and source contacts. The WS_2_ phototransistors can be operated in self-powered mode, yielding both a current and a voltage when exposed to light. The spectral photoresponse in the visible and the NIR ranges shows a high responsivity (4.5 μA/W) around 1250 nm, making the devices promising for telecommunication applications.

## Introduction

Among van der Waals layered materials, transition metal dichalcogenides (TMDs) have recently gained attention from the scientific community for their peculiar optoelectronic characteristics [1–5]. Indeed, TMDs exhibit a unique combination of atomic-scale thickness, direct bandgap, strong spin–orbit coupling and favorable electronic and mechanical properties [6–9], which make them interesting for fundamental studies as well as for applications in energy harvesting [10, 11], optoelectronics [12–17], spintronics [18], data storage [19, 20], synaptic devices [21, 22], flexible devices [23–27], etcetera.

Tungsten disulphide (WS_2_) belongs to the family of TMDs and presents a layered hexagonal crystal structure, with layers held together by van der Waals forces. Due to the weak interlayer interactions, WS_2_ crystals can be easily exfoliated mechanically by scotch tape [28, 29]. The WS_2_ monolayer consists of three atomic planes in which the W atomic plane is sandwiched between two planes of S atoms, forming the S-W-S structure, and has a thickness of ~ 0.625 nm [30]; moreover, it is free from dangling bonds, with stable and nonreactive surface [31].

Like in other semiconducting TMDs, the WS_2_ band structure depends on the number of layers. The 1.3 eV bandgap of the bulk widens up to 2.1 eV in the monolayer [32] and a transition from indirect to direct bandgap is observed [33]. The direct bandgap enhances the photoluminescence (PL) that in the monolayer is more than three orders of magnitude stronger than in the bulk [34, 35]. Conversely, multilayer WS_2_ is advantageous for photodetection due to increased optical absorption and carrier mobility [36].

The excellent electronic and optical properties of WS_2_ have already been exploited in optoelectronic devices [9, 37, 38] like photodetectors, LEDs, lasers, and optical modulators [39–45]. Following the trend, in the present work, multilayer WS_2_ flakes are used to fabricate broadband phototransistors over Si/SiO_2_ substrates. The transistors show excellent field-effect properties with a high electron mobility of 6 cm^2^/Vs. The devices are tested under a laser light source, which can be tuned in wavelength and power. The extensive photocurrent spectroscopy analysis, performed in the visible (500–700 nm) and near-infrared (1100–1300 nm) range, shows that the devices are promising for optical telecommunications.

## Materials and methods

Several WS_2_ flakes were transferred by mechanical exfoliation onto a highly doped (resistivity $\rho = 0.001$ Ω cm) p-type Si/SiO_2_ substrate, with oxide thickness of 85 nm. Then, two Ti/Au (10/100 nm) pads were deposited on top of selected flakes as electrical contacts.

**Fig. 1:**
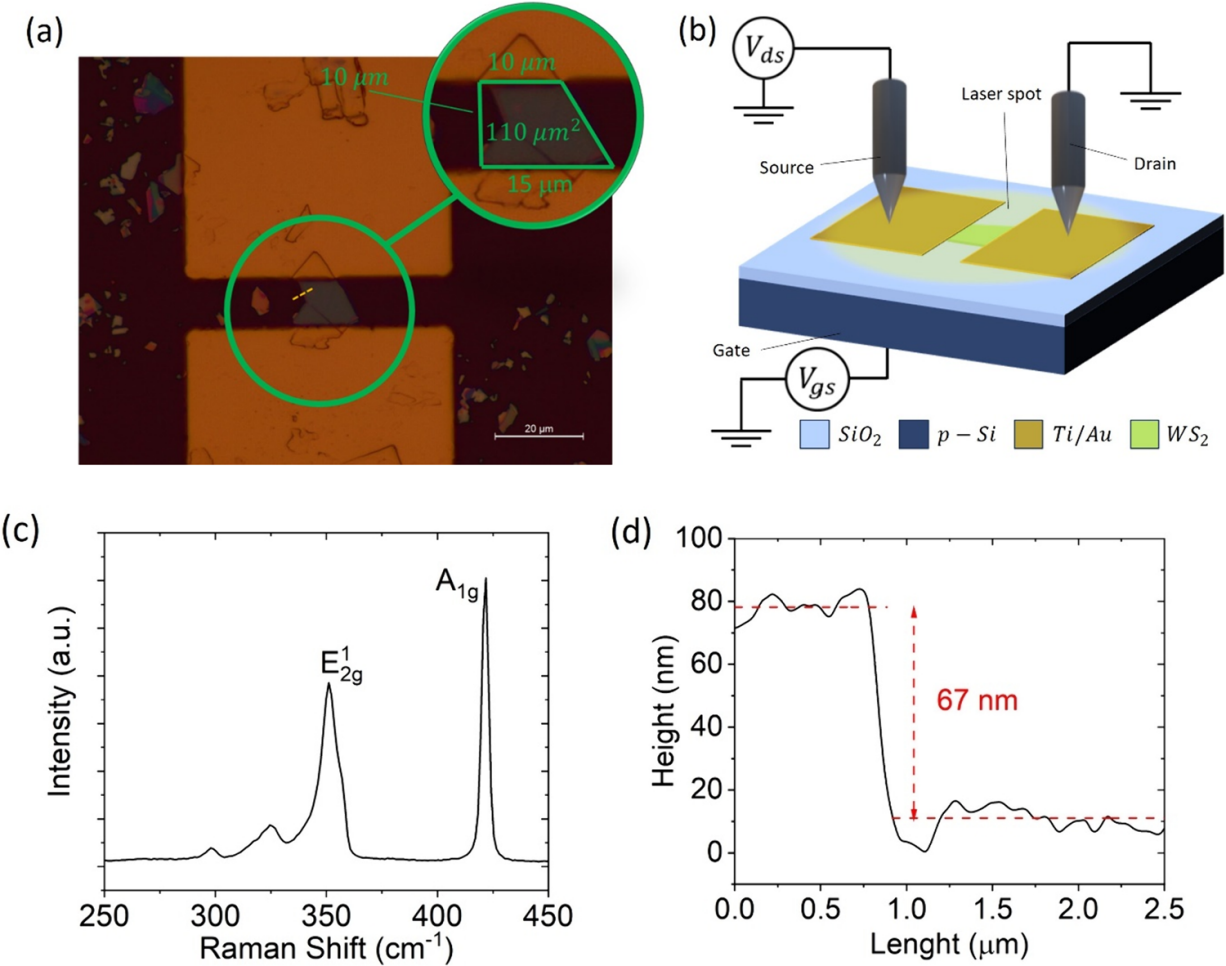
**a** Optical image of the device under investigation in this study. The area of the flakes is estimated in the green circle to be around 110 μm^2^. **b** Device and measurements setup schematic. **c** Raman spectroscopy. **d** AFM profile of the device

Figure 1a shows an optical image of the device that will be considered in the following. The magnification (green circle) displays the WS_2_ flake (highlighted by the green trapezoid), with an optical active area $A_{act} \approx 110$ μm^2^. Figure 1b presents a schematic of a device and the measurement experimental setup, pointing out that the laser spot (with area $A_{spot} \approx 0.13\;{\text{mm}}^{2}$), covers the entire device. The Si substrate is the back gate, while the two Ti/Au pads are the source and drain of the transistor. Figure 1c shows the Raman spectrum of the WS_2_ flake, measured at room temperature by means of a commercial inVia Renishaw Raman microscope with a Centrus CCD detector with 514 nm laser excitation. The Raman spectrum demonstrates multiple distinct peaks, each corresponding to specific vibrational mode of the crystal lattice. The Raman scattering peaks of the WS_2_ flake are located at 421.78, 351.06, 324.67, and 297.58 cm^−1^, consistent with the reported data [46].

Figure 1d shows the atomic force microscope (AFM) image of the device and the height profile along the line crossing the border of the WS_2_ flake (yellow dashed line in Fig. 1a). The step height indicates a thickness of 67 nm, corresponding to about 100 layers.

To get insight into the atomistic structure and electrical characteristics of WS_2_ in contact with Ti metal, density functional theory (DFT) and nonequilibrium Green’s function (NEGF) have been carried out as implemented in QuantumATK [47, 48]. Linear combination of numerical atomic-orbital basis set and generalized gradient approximation (GGA) norm-conserving pseudopotentials from PseudoDojo with medium basis sets [49] are employed in the simulations. Brillouin zone integrations are performed over a grid of k-points generated according to the Monkhorst–Pack scheme [50], with a density of approximately 10 k-points per angstrom. An energy cut-off of 110 Ha is considered for the discretized grid, and all structural relaxation is performed with a maximum force of less than 0.02 eV Å^−1^. For discretized grid, ∼ 880 k-point per angstrom have been used for the Green’s function calculations, in the direction normal to the Ti/WS_2_ interface plane. The metal crystallographic orientation with common supercell with minimal strain for Ti[0001] has been considered for commensurable interface leading to 3.8% strain. The strain arising from the lattice constant mismatch between the materials at the Ti/WS_2_ interface has been applied to the metal electrodes, since, unlike WS_2_, a few percent in-plane lattice constant of metal surface does not affect its electronic properties considerably [51].

## Results and discussion

**Fig. 2:**
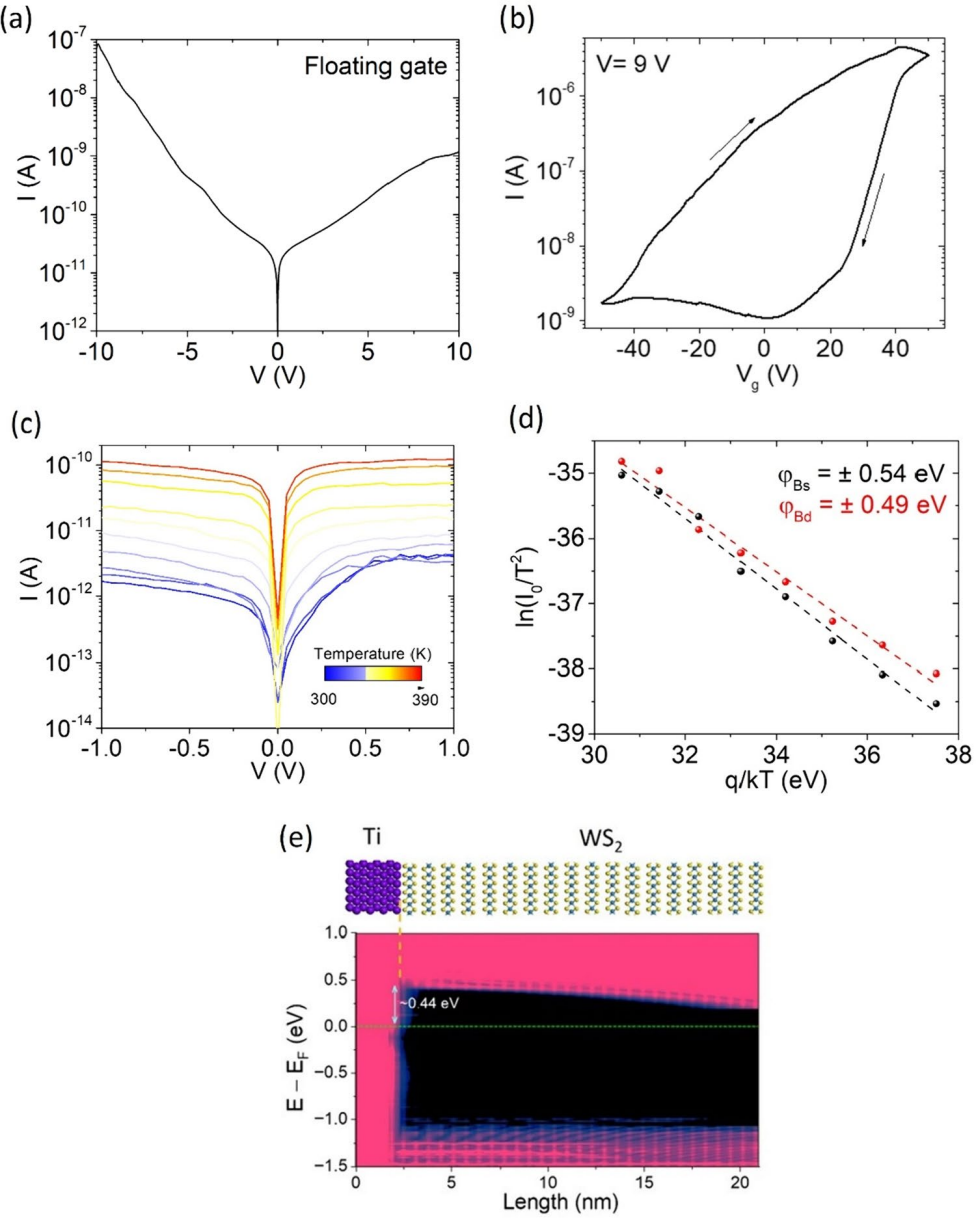
**a** Current–voltage characteristic with floating gate in dark condition. **b** Transfer characteristic at V = 9 V in dark condition. **c** Current–voltage characteristics as a function of the temperature. **d** Evaluati**a**on of the Schottky barrier heights formed under drain and source contacts. **e** Atomic structure (top) and local density of states (LDoS) of the Ti/WS_2_ interface (bottom), demonstrating the band offset at the interface

Figure 2a shows the current–voltage (I–V) characteristic of the device under ambient dark condition and with floating gate (i.e. gate electrode not connected to the source-measurement unit). The slightly asymmetric I–V curve suggests the presence of two slightly asymmetric Schottky barriers under drain and source contacts [52]. The drain current (I) as a function of the gate-source voltage (V_g_), i.e. the transfer characteristic, for − 50 V < V_g_ < 50 V and fixed source-drain voltage (V =  9 V), is reported in Fig. 2b. The transfer characteristic shows an n-type behavior. A wide hysteresis, typical of 2D materials-based transistors and due to intrinsic and extrinsic charge trapping in the channel region [53–57], is observed in the transfer characteristic. The low value of the drain current measured in Fig. 2a indicates that the device was initially on the lower branch of the hysteretic loop. From the forward sweep, the electron mobility can be extracted as:

$$\mu = \left( {\frac{{dI_{d} }}{{dV_{g} }}} \right)\frac{L}{CW}\frac{1}{V} = 6 \;{\text{cm}}^{2} \;{\text{V}}^{ - 1} \;{\text{s}}^{ - 1}$$

where L and W are the length and the width of the channel, and C is the oxide capacitance per unit area ($C = 39\;{\text{nF/cm}}^{2}$). Besides, as another figure of merit of the transistor, the subthreshold swing, defined as $SS = \left( {\frac{{d\left( {\ln \left( I \right)} \right)}}{{dV_{g} }}} \right)^{ - 1}$, is evaluated to be $6.5\;{\text{V/dec}}$.

For a deeper understanding of the transport phenomena, we measured the device I–V characteristics at different temperatures, from 300 to 390 K. Figure 2c show these I–V curves in dark. The device shows that the current increases for increasing temperature, pointing to a conduction dominated by thermionic emission. Indeed, the two Schottky barriers at the contacts make the device behave as two back-to-back diodes. This means that the current is the reverse saturation current of a Schottky diode for both negative and positive drain voltages. Hence, the current is low and strongly dependent on temperature. We can estimate the Schottky barrier heights (SBHs, $\varphi_{B}$) formed at the Ti/WS_2_ interface from the current versus temperature behaviour at given drain voltages (here, V =  ± 0.5 V). The Schottky diode reverse saturation current is expressed as:

$$I_{0} = SA^{**} T^{2} e^{{ - \varphi_{B} /k_{b} T}}$$

where S is the area of the junction, A** is the effective Richardson constant, ${k}_{b}$ is the Boltzmann constant and T is the temperature. The above equation can be rewritten as:

$$\ln \left( {I_{0} /T^{2} } \right) = \ln \left( {SA^{**} } \right) - \varphi_{B} /k_{b} T$$

from which we can evaluate the SBHs as the slope of the data plotted in Fig. 2d. As expected, the SBHs formed at the drain contact ($\varphi_{Bd} = 0.49\;{\text{eV}}$) and at the source contact ($\varphi_{Bs} = 0.54\;{\text{eV}}$) do not differ significantly. The SBH of a metal/semiconductor Schottky junction can be compared with the local density of states (LDoS) along the transport direction [58]. The experimentally extracted SBH at the Ti/WS_2_ interface is consistent with our DFT simulations, where a potential barrier of ~ 0.44 eV is obtained at the interface (Fig. 2e).

**Fig. 3:**
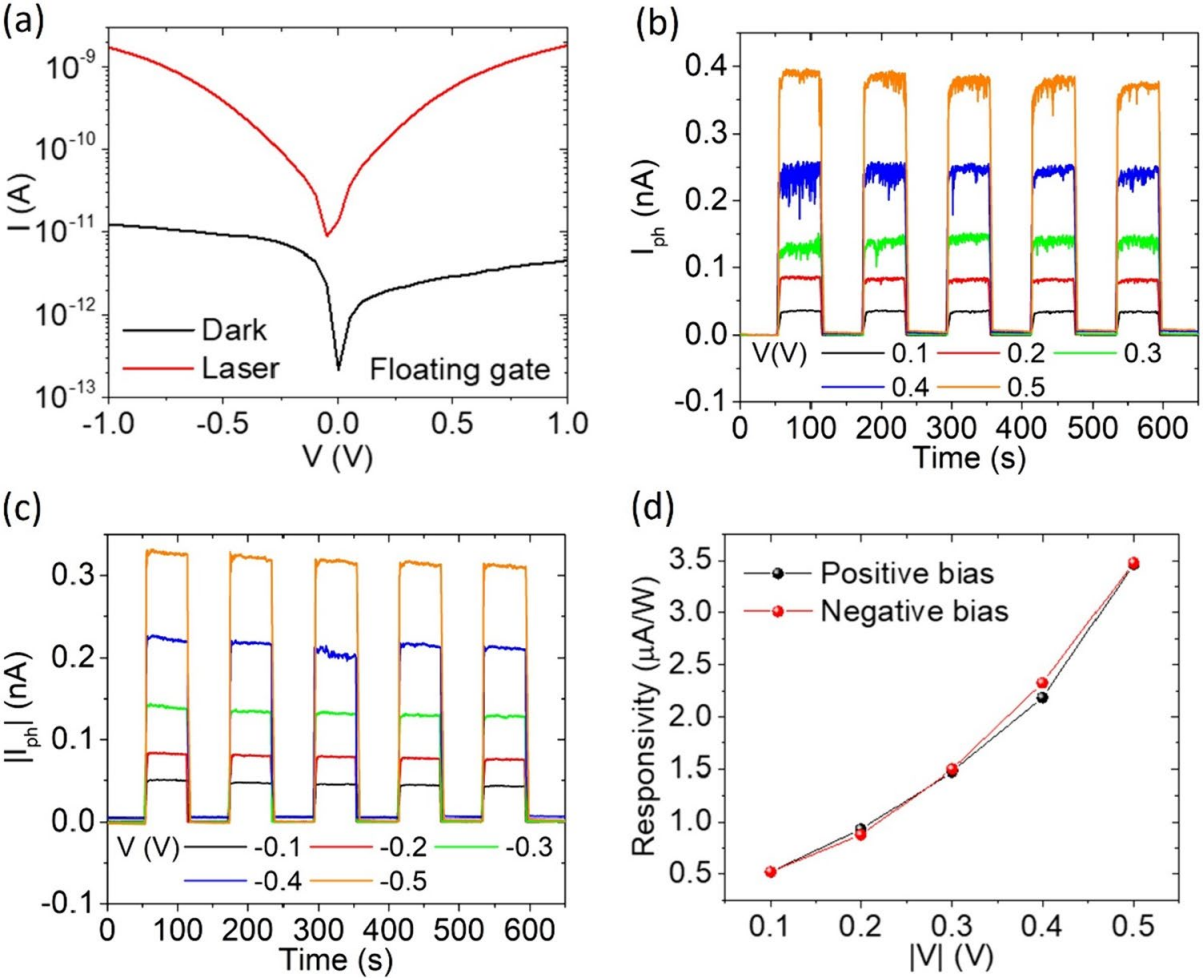
**a** Current–voltage characteristic with floating gate in dark (black line) and under laser irradiation. **b**, **c** Photocurrent peaks and **d** responsivity at different bias voltages

To investigate the photoresponse of the device we exposed it to a white laser source. The I–V characteristics in the dark (black line) and under laser irradiation (red line) are displayed in Fig. 3a. The low value of the dark drain current corresponds to the lower branch of the hysteretic loop (Fig. 2b) and indicates fully depleted channel. When exposed to light, the device shows an increased current due to the photoconduction effect. Besides, a left shift is presented under laser irradiation, suggesting that also a photovoltaic effect occurs [59, 60].

As a first approach to the study of the optoelectronic properties of the device, a series of white laser pulses (60 s long) have been sent on the sample. Figure 3b, c show the photoresponse peaks at several applied voltage biases, ranging from – 0.5 V to 0.5 V, with steps of 0.1 V. An increasing photocurrent ($I_{ph} = I_{light} {-} I_{dark}$) is observed for increasing bias voltages.

The responsivity (R), which is the most remarkable figure of merit of a photodetector, is defined as the photogenerated current per unit of incident optical power,

$$R = I_{ph} /P_{inc} \left( \lambda \right)$$

where $$P_{inc} \left( \lambda \right) = P\left( \lambda \right){* }A_{act} /A_{spot}$$.

The responsivity of the device versus the applied voltage bias is displayed in Fig. 3d. The responsivity shows an increase for increasing drain bias. The voltage dependence of the responsivity is due to enhanced exciton dissociation into free electrons and holes [61] and image-force lowering of the Schottky barrier caused by the increasing in-plane electric field [62, 63].

**Fig. 4:**
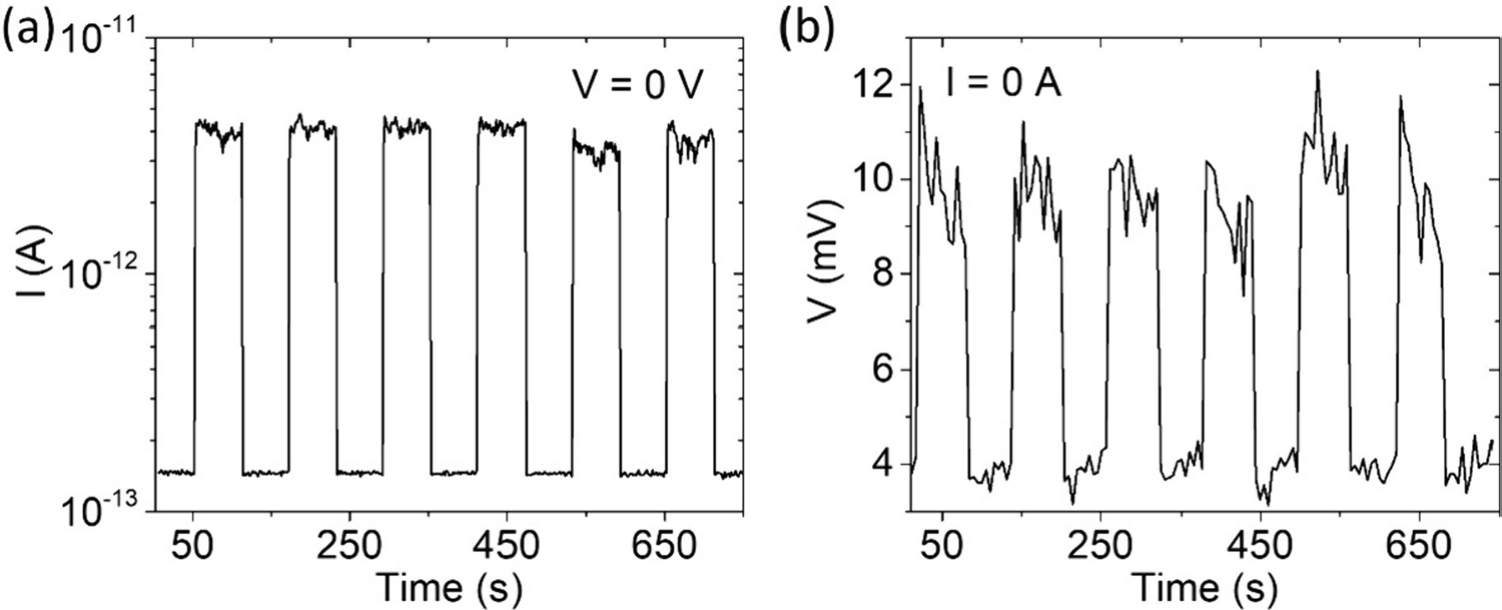
**a** Photocurrent at 0 V and **b** photovoltage at 0 A peaks obtained by 60 s laser pulses

We completed the investigation of the device by testing its operation in self-powered mode. We irradiated the sample with a series of laser pulses (60 s long) without voltage bias (Fig. 4a) or current bias (Fig. 4b). The WS_2_ transistor shows a photogenerated current $I_{ph} \approx 4\;{\text{pA}}$ at V = 0 V and photogenerated voltage $V_{ph} \approx 8\;{\text{mV}}$ at I = 0 A, enabling its use as a self-powered device. The obtained zero-bias photoresponse, which is defined as $\frac{{I_{light} - I_{dark} }}{{I_{dark} }}$, is $\approx$ 2700%.

**Fig. 5:**
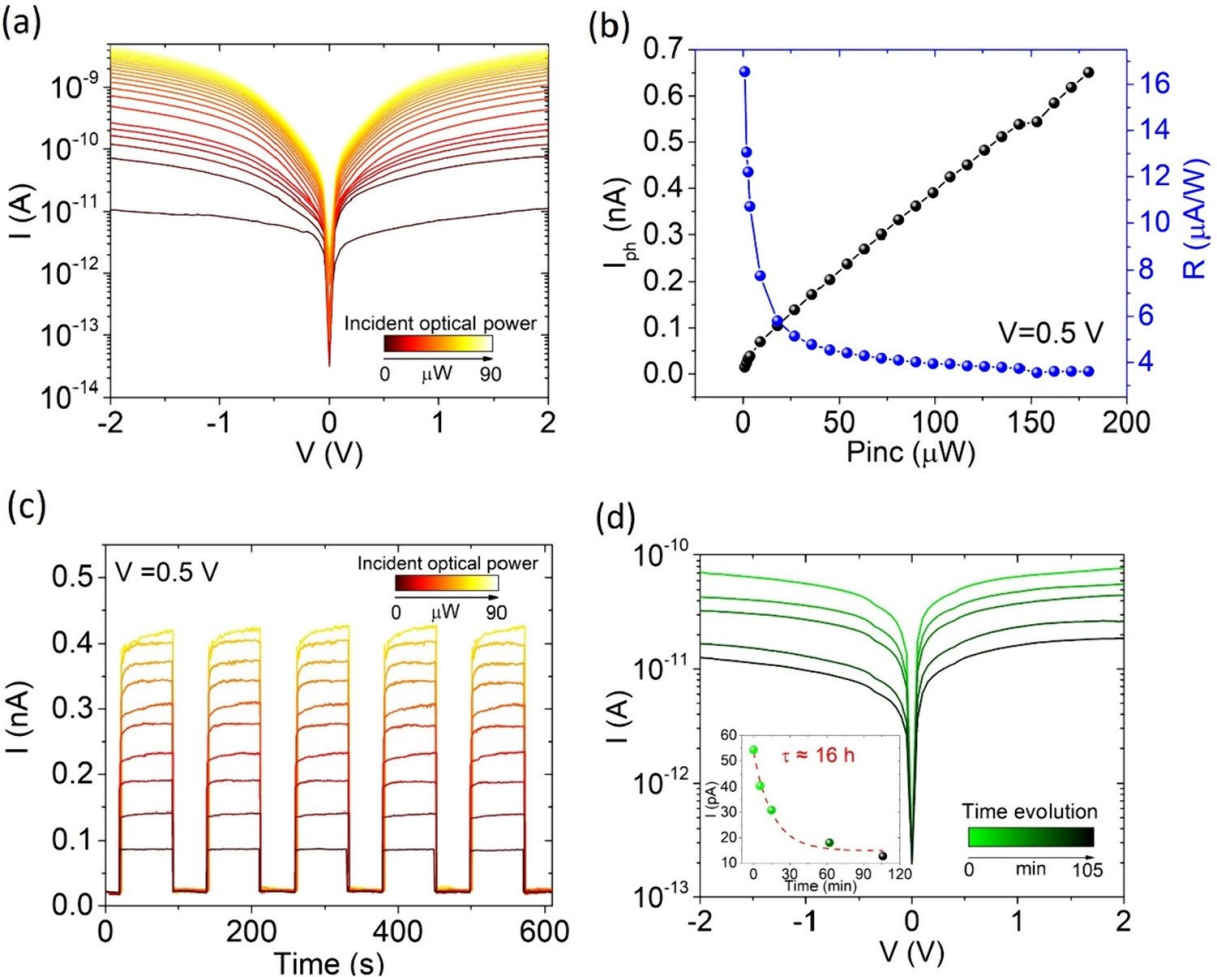
**a** I–V characteristics at different incident optical powers. **b** Photocurrent and responsivity of the device versus incident optical power. **c** Current peaks at different incident optical power. **d** I–V characteristics in dark measured at successive times. Inset: time evolution of the current extracted at V = 0.5 V

To further understand the photodetection properties of the device, we measured I–V curves at different incident optical powers (Fig. 5a). Figure 5b reports higher responsivity at lower incident power, along with a photocurrent that changes with P_inc_, following a sub-linear behaviour. The increasing incident optical power leads to an increase in photogenerated charge carriers, which corresponds to an increase in photocurrent. The responsivity, instead, decreases at high incident optical power, when the enhanced carrier density leads to an increase of scattering rate favouring electron–hole recombination and limiting the charge carrier mobility [64, 65].

The peaks in Fig. 5c show a photocurrent which increases with increasing P_inc_. The photocurrent is due to excitations of electrons from trap states and to the presence of adsorbates, such as O_2_ and H_2_O, on the WS_2_ surface, which are desorbed under light exposure with consequent increase of the WS_2_ n-doping level [66].

The graph in Fig. 5d shows the time evolution of the dark current over a time of about 100 min. The inset of Fig. 5d reports the extracted dark current at V = 0.5 V, which can be fitted by an exponential law, giving a time constant of about 16 h. The slow decay is dominated by the slow photocharge trapping and the adsorption of O_2_ and H_2_O. The device presents a persistent photoconductivity, which can make it suitable for photonic neuromorphic devices [67].

**Fig. 6:**
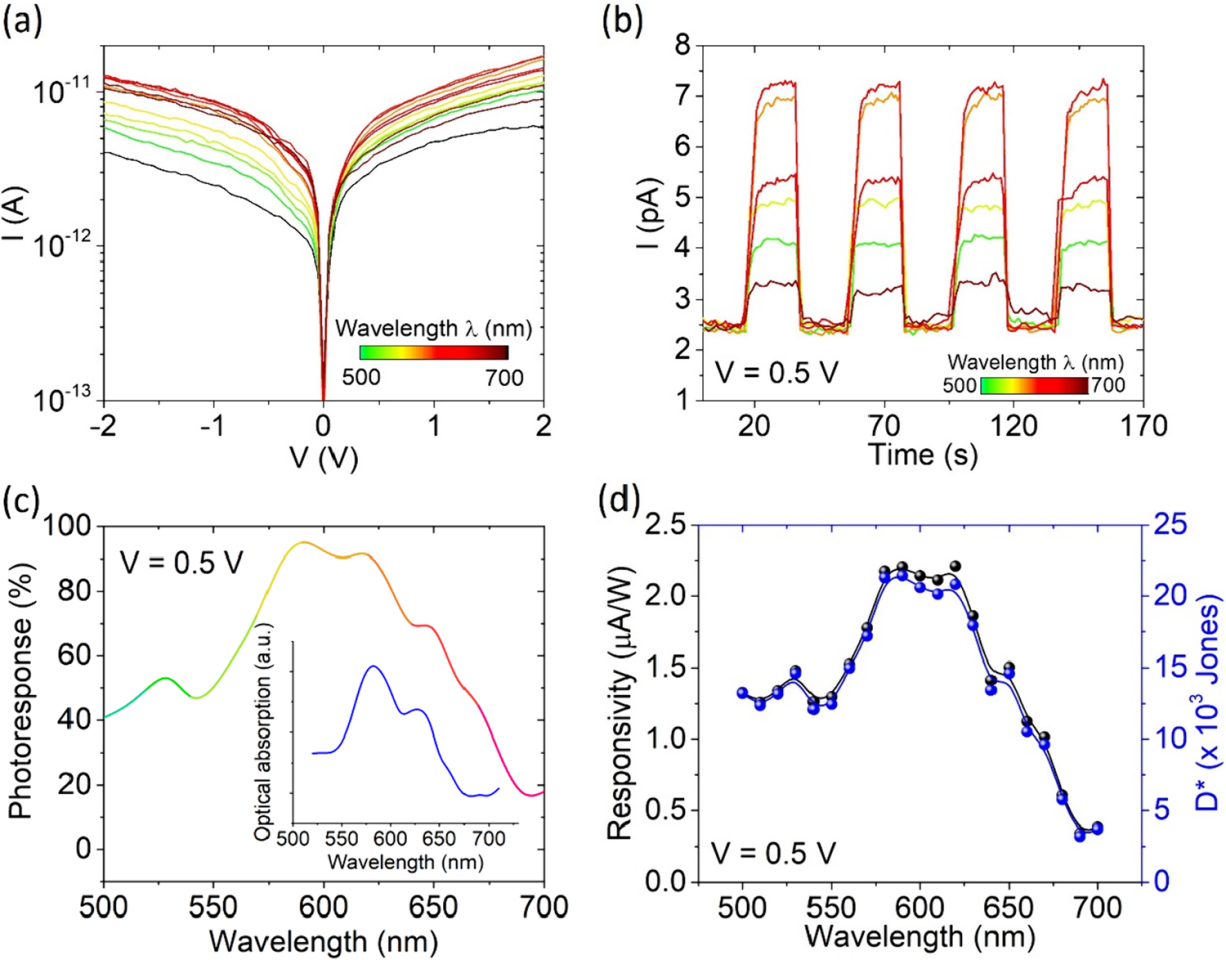
**a** I–V characteristics and **b** current peaks varying wavelength in the visible range. **c** Photoresponse, **d** responsivity and external quantum efficiency of the device over the visible wavelength range (500–700 nm). Inset of **c** reports the measured absorption spectrum in the visible wavelength range

We also investigated light absorption in the visible and near-infrared (NIR) range. Figure 6a reports I–V characteristics at different wavelengths, from 500 to 700 nm. Furthermore, at given wavelengths, the device was exposed to a series of light pulses yielding the current peaks displayed in Fig. 6b.

The spectral photoresponse reveals a peak at about $\lambda = 590\;{\text{nm}}$, corresponding to a photon energy of $E_{g} = 2.1\;{\text{eV}}$ (Fig. 6c). Few layers WS_2_ shows an indirect band-gap of 1.4 eV and a direct band-gap of 2.1 eV [68], which explains the observed behaviour. The inset of Fig. 6c shows the measured optical absorption spectrum that matches the spectral photoresponse in the visible region [69, 70]. The device shows a responsivity up to $R = 2.2$ μA/W, along with a specific detectivity [71], defined as $D* = \sqrt {\frac{A}{{2qI_{dark} }}} R$, of about $2 \cdot 10^{4} {\text{Jones}}$ (Fig. 6d).

**Fig. 7:**
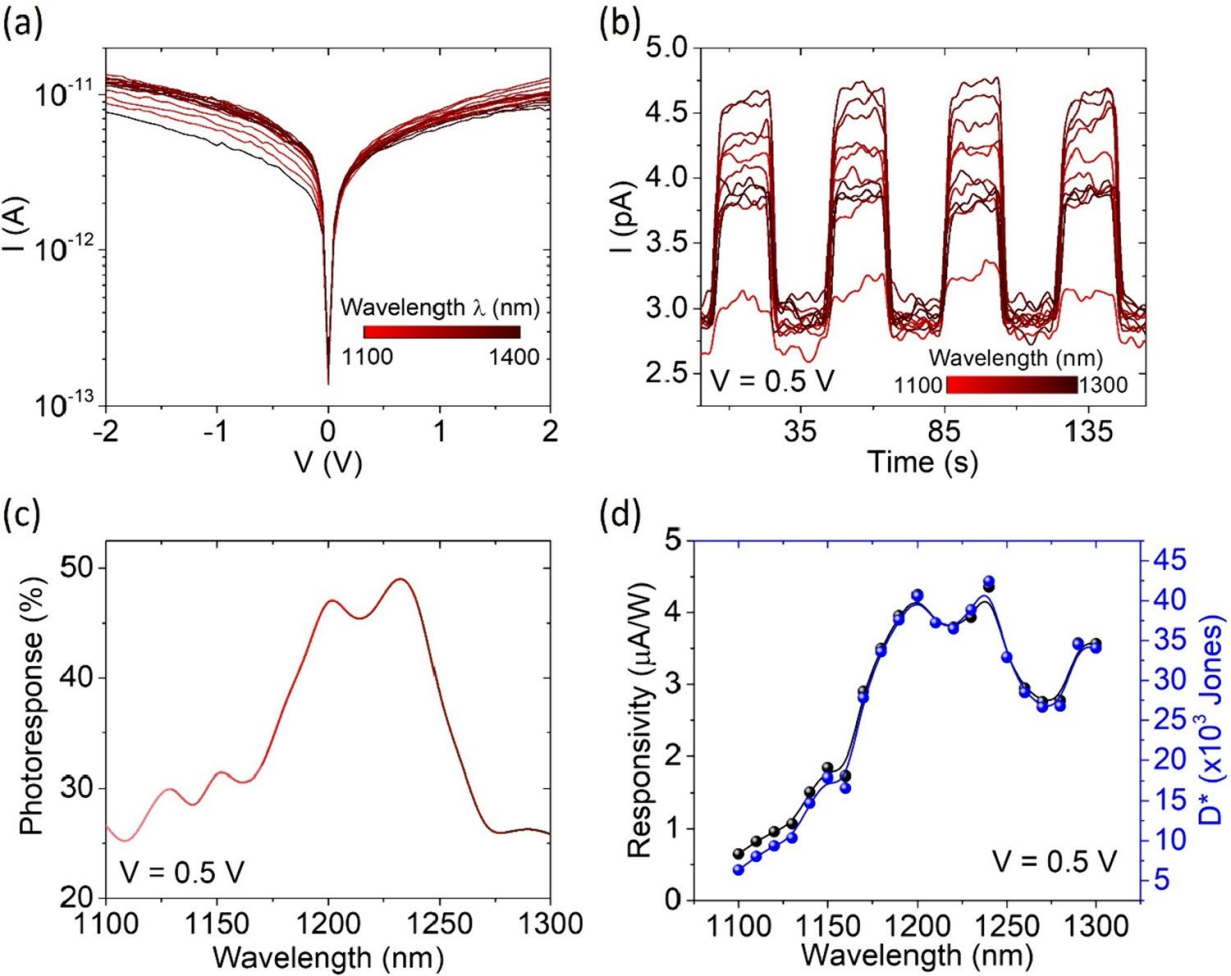
**a** I–V characteristics and **b** current peaks at different wavelengths in the NIR range. **c** Photoresponse, **d** responsivity and external quantum efficiency of the device over the wavelength NIR range (1100–1300 nm)

A similar analysis has been conducted over the NIR range. Figure 7 reports IV characteristics, current peaks, photoresponse, responsivity and specific detectivity at different wavelengths from 1100 to 1400 nm. The absorption spectrum presents the highest peak at about 1250 nm, where the responsivity reaches 4.5 μA/W and D* is $4 \cdot 10^{4} \;{\text{Jones}}$. While the literature does not provide a definitive explanation, the emergence of a peak in the near-infrared region may be attributed to the presence of interlayer excitons. These excitons can exhibit absorbance at lower energies, particularly when the active medium consists of several distinct multilayer flakes [72–74]. Indeed, our channel appears to be made of two (or even more) different flakes, as shown in the optical image of Fig. 1a. The high responsivity in the NIR region makes the device a promising candidate for applications in optical telecommunications.

## Conclusions

Field effect transistors based on mechanically exfoliated multilayer WS_2_ have been studied as photodetectors. Their operation in self-powered mode has been investigated, revealing photo-signals at zero voltage or current biases. Indeed, the WS_2_ device shows a photogenerated current $I_{ph} \approx 4\;{\text{pA}}$ at V = 0 V and photogenerated voltage $V_{ph} \approx 8\;{\text{mV}}$ at I = 0 A. Besides, a complete optoelectronic study of the device as a function of the incident laser power has been conducted, revealing higher performances at low incident power. Finally, we studied the photoresponse of the device in both visible and infrared regions, reporting a high responsivity at 1250 nm, paving the way for WS_2_ applications in optical telecommunication.

## Data Availability

Data sets generated during the current study are available from the corresponding author on reasonable equest.
